# Cymbopogon Proximus Essential Oil Protects Rats against Isoproterenol-Induced Cardiac Hypertrophy and Fibrosis

**DOI:** 10.3390/molecules25081786

**Published:** 2020-04-13

**Authors:** Hassan N. Althurwi, Maged S. Abdel-Kader, Khalid M. Alharthy, Mohamad Ayman Salkini, Faisal F. Albaqami

**Affiliations:** 1 Pharmacology and Toxicology Department, College of Pharmacy, Prince Sattam bin Abdulaziz University, AlKharj 11942, Saudi Arabia; k.alharthy@psau.edu.sa (K.M.A.); f.albaqami@psau.edu.sa (F.F.A.); 2 Pharmacognosy Department, College of Pharmacy, Prince Sattam bin Abdulaziz University, AlKharj 11942, Saudi Arabia; mpharm101@hotmail.com (M.S.A.-K.); m.salkini@psau.edu.sa (M.A.S.); 3 Department of Pharmacognosy, College of Pharmacy, Alexandria University, Alexandria 21215, Egypt

**Keywords:** cardiac fibrosis, cardiac hypertrophy, cardiac remodeling, *Cymbopogon proximus*, isoproterenol

## Abstract

Cardiac hypertrophy is an independent risk factor of many cardiovascular diseases. Several cardiovascular protective properties of *Cymbopogon proximus* have been reported. However, no reports investigating the direct effect of *C. proximus* essential oil on the heart are available. The goal of this study was to explore the cardioprotective effect of *C. proximus* on cardiac hypertrophy and fibrosis. Male albino rats were administered *C. proximus* essential oil in the presence or absence of hypertrophic agonist isoproterenol. Cardiac hypertrophy and fibrosis were assessed using real-time polymerase chain reaction (PCR) and histological examination. Pre- treatment of rats with *C. proximus* decreased the ratio of heart weight to body weight and gene expression of hypertrophy markers atrial natriuretic peptide (ANP), brain natriuretic peptide (BNP), and β-myosin heavy chain (β-MHC), which were induced by isoproterenol. Moreover, *C. proximus* prevented the increase in gene expression of fibrosis markers procollagen I and procollagen III and alleviated the collagen volume fraction caused by isoproterenol. The pre- treatment with *C. proximus* essential oil conferred cardio-protection against isoproterenol- induced cardiac hypertrophy and fibrosis.

## 1. Introduction

Globally, cardiovascular diseases (CVDs) remain the leading cause of mortality and morbidity [1]. Although advances have been made in cardiovascular research, CVDs are still responsible for 31% of all deaths worldwide [2]. In general, cardiac diseases are viewed as a chain of events known as the CVD continuum, which if untreated, eventually leads to heart failure (HF) and sudden death [3,4]. Currently, more than 26 million patients have been diagnosed with HF [5]. The prognosis for patients with HF remains poor with 50% of patients dying within five years of diagnosis [6]. Cardiac hypertrophy, a thickening of the heart wall in response to increased cardiac stress, occurs early on in the CVD continuum and is considered as a compensatory response that permits normal cardiovascular function at rest [7,8]. However, prolonged hypertrophy is now recognized as a credible surrogate endpoint of HF and a major risk factor for heart disease, including coronary artery disease (CAD), arrhythmia, and hypertension [9]. Therefore, studying cardiac hypertrophy is important to identify new therapeutic options that could prevent and/or treat CVDs in the early stages.

Cymbopogon, a genus of plants with many species known for their high essential oil content, are widely distributed throughout the tropical and subtropical regions of Asia, Africa, and America [10]. The use of *Cymbopogon* species in traditional medicine is well documented. Several illnesses, such as coughs, fever, infections, cancer, and digestive disorders, have reportedly been treated using various species of *Cymbopogon* worldwide [10,11]. Moreover, in vivo and in vitro studies have revealed beneficial pharmacological effects of *Cymbopogon* spp., including anticancer, cardioprotective, anti-inflammatory, antioxidant, antidiabetic, anticholinesterase, antibacterial, and antifungal properties [12,13,14,15,16,17,18].

One species of interest is *C. proximus* (common names: Halfabar or Maharaib), a strongly aromatic common grass widely distributed in parts of Southern Egypt and Northern Sudan. *C. proximus* has been used for several decades by the inhabitants of these regions as a diuretic and antispasmodic agent due to its potent ability to induce smooth-muscle relaxation [19]. *C. proximus* reportedly possesses many biological properties, including hypoglycemic, antipyretic, bronchodilation, antibacterial, anticonvulsant, and antiemetic activities [15,20,21,22,23]. Interestingly, *C. proximus* has been shown to exhibit a hypotensive effect in normotensive rats and protect against nitro-L-arginine methyl ester (L-NAME)-induced hypertension [23,24]. At the cellular level, extracts of *C. proximus* have been shown to have antioxidant, anti-inflammatory, and antiapoptotic properties [20,22]. These findings indicate that *C. proximus* may be a potential protective agent against cardiac diseases. To the best of our knowledge, no research has evaluated the effect of *C. proximus* against cardiac remodeling with a focus on myocardial hypertrophy and fibrosis. We hypothesized that *C. proximus* essential oil would confer cardioprotection against isoproterenol-induced cardiac hypertrophy and fibrosis. The findings from this research may provide the first evidence for the potential use of *C. proximus* as a modulator of cardiac remodeling. This is especially important with the current need to identify new alternative medicines that are natural and safe.

## 2. Results

### 2.1. Gas Chromatography–Mass Spectrometry (GC-MS) Analysis

Analysis of the *C. proximus* essential oil by GC-MS identified forty different chemical constituents, which represented 93.27% of the total oil extract. The main components included elemol (23.54%), piperitone (19.86%), α-eudesmol (7.63%), and β-eudesmol (11.35%), which together comprised 62.38% of the oil. A complete listing of the GC-MS results is shown in Table 1.

### 2.2. Effect of *C. Proximus* Oil and/or Isoproterenol on Body and Heart Weights

**Figure 1:**
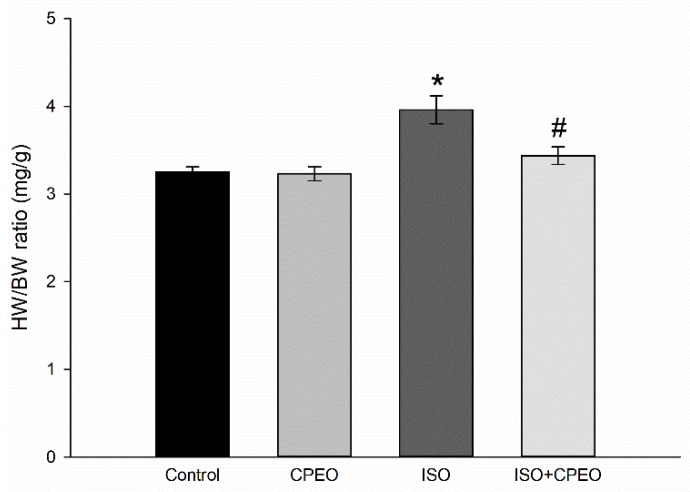
Effect of *Cymbopogon proximus* essential oil (CPEO) on body weight (BW) and heart weight (HW) of rats. Male albino rats were injected intraperitoneally daily with vehicle (saline + olive oil) as the control, CPEO (800 µL/kg/d), isoproterenol (ISO; 5 mg/kg), or CPEO (800 µL/kg/d) plus isoproterenol (5 mg/kg). CPEO administration was started 4 d prior to isoproterenol administration and continued concurrently thereafter for an additional 3 d. The HW/BW ratio (mg/g) was determined for each animal after 7 d of treatment with vehicle, CPEO, ISO, or a combination of ISO+CPEO. The results are presented as the means of six independent experiments ± SEM. * *p* < 0.05 compared to control, # *p* < 0.05 compared to ISO-treated rats.

Isoproterenol treatment caused a significant increase of 23% in the ratio of heart weight to body weight (HW/BW) compared with that of the control group (*p* < 0.001). On the other hand, rats pretreated with *C. proximus* oil displayed a 69% reduction in the isoproterenol-mediated increase of HW/BW compared with that of the isoproterenol group (*p* = 0.017). Furthermore, no significant difference in HW/BW was found between the control group and the group treated with *C. proximus* oil alone (Figure 1).

### 2.3. Effect of *C. Proximus* Oil and/or Isoproterenol on Hypertrophy Markers

**Figure 2:**
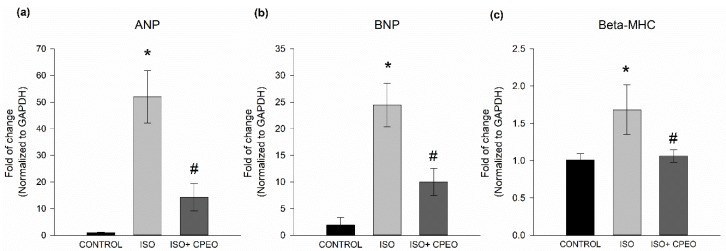
Effect of *Cymbopogon proximus* essential oil (CPEO) and/or isoproterenol (ISO) on hypertrophy markers. Male albino rats were injected intraperitoneally daily with vehicle (Control), PEO (800 µL/kg/d), ISO (5 mg/kg), or CPEO (800 µL/kg/d) plus ISO (5 mg/kg). Oil administration was started 4 d prior to ISO administration and continued concurrently thereafter for an additional 3 d. Expression of hypertrophic genes ANP (**a**), BNP (**b**), and β-MHC (**c**) in heart tissue based on mRNA levels measured using quantitative real-time polymerase chain reaction. The results are presented as the means of six independent experiments ± SEM. * *p* < 0.05 compared to control, # *p* < 0.05 compared to ISO-treated rats.

In addition to increased heart mass, pathological cardiac hypertrophy is characterized by the activation of the fetal gene program, thereby changing the expression of different genes including ANP, BNP, and β-MHC. Thus, the expression of these genes is one of the most consistent markers of pathological cardiac hypertrophy [25,26,27]. To investigate whether *C. proximus* oil and/or isoproterenol treatment altered the expression level of hypertrophy markers, we measured cardiac expression of ANP, BNP, and β-MHC. Isoproterenol alone caused significant induction of ANP, BNP and β-MHC expression with mRNA levels increasing 52-fold (*p* < 0.001), 12.5-fold (*p* < 0.001), and 0.7-fold (*p* = 0.02), respectively (Figure 2). However, relative to those in isoproterenol-treated rats, pretreatment with *C. proximus* oil significantly decreased the isoproterenol-mediated induction of ANP, BNP and β-MHC by 73% (*p* = 0.004), 59% (*p* = 0.007), and 91% (*p* = 0.024), respectively (Figure 2).

### 2.4. Effect of *C. Proximus* Oil and/or Isoproterenol on Myocardial Architecture

**Figure 3:**
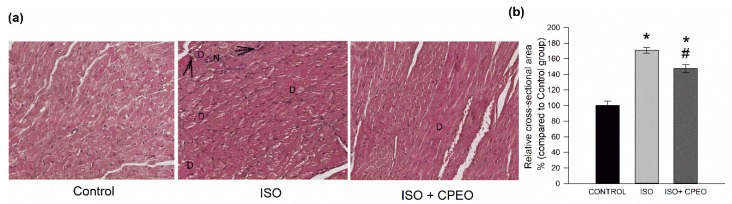
Effect of *Cymbopogon proximus* essential oil (CPEO) and/or isoproterenol (ISO) on myocardial architecture. Male albino rats were injected intraperitoneally daily with vehicle (Control), CPEO (800 µL/kg/d), ISO (5 mg/kg), or CPEO (800 µL/kg/d) plus ISO (5 mg/kg). Oil administration was started 4 d prior to ISO administration and continued concurrently thereafter for an additional 3 d. Histological examination and pathological changes in heart tissue. (**a**) Representative images of hematoxylin and eosin (H&E)-stained fields are shown for the left ventricles of rats of the control group and rats treated with ISO+CPEO or ISO alone (magnification, × 200), (necrosis (N); degeneration (D); pyknotic changes in the nuclei (arrows)) (**b**) Mean cross-sectional areas of cardiomyocytes from left ventricles of rats from the indicated experimental groups were calculated and are shown. The results are presented as the means of four independent experiments ± SEM. * *p* < 0.05 compared to control, # *p* < 0.05 compared to ISO-treated rats.

Histopathological examination of cardiac tissue sections from the control group revealed typical cell distribution and normal myocardium architecture, demonstrating variable fiber diameters and central positions of the nuclei. However, examination of cardiac tissue sections from isoproterenol- treated rats revealed moderate cardiomyocyte degeneration, necrosis, pyknosis, and a 71% increase in cross-sectional area of cardiac myocytes cells compared to that of the control group (*p* < 0.001). Pretreatment with *C. proximus* oil resulted in a less severe necrosis and a 33% decrease in cross-sectional area of cardiac myocytes compared to that of the isoproterenol group (*p* = 0.005; Figure 3). However, the pretreatment with *C. proximus* oil did not restore this response to the control levels (*p* < 0.001).

### 2.5. Effect of *C. Proximus* Oil and/or Isoproterenol on Myocardial Fibrosis

**Figure 4:**
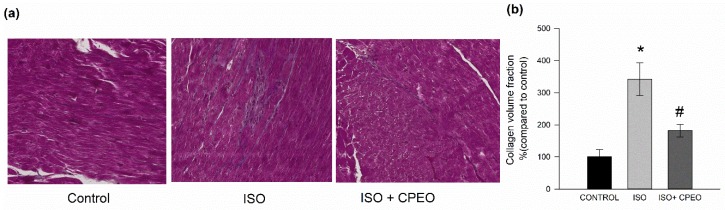
Effect of *Cymbopogon proximus* essential oil (CPEO) and/or isoproterenol (ISO) on myocardial fibrosis. Male albino rats were injected intraperitoneally daily with vehicle, CPEO (800 µL/kg/d), ISO (5 mg/kg), or CPEO (800 µL/kg/d) plus ISO (5 mg/kg). Oil administration was started 4 d prior to ISO administration and continued concurrently thereafter for an additional 3 d. Histological analysis and pathological changes in cardiac tissue. (**a**) Representative Masson’s trichrome staining of left ventricles of control group rats and rats treated with ISO+CPEO or ISO alone. (**b**) Quantitative analysis of myocardial collagen volume fraction (CVF) in the left ventricles of rats of the experimental and control groups. The results are presented as the means of four independent experiments ± SEM. * *p* < 0.05 compared to control, # *p* < 0.05 compared to ISO-treated rats.

To assess the degree of myocardial fibrosis in response to *C. proximus* oil and/or isoproterenol, heart sections were stained with Masson’s trichrome and the percentages of fibrotic tissue in the images were determined using ImageJ software. Collagen volume fraction (CVF) values in the isoproterenol-treated group increased 242% compared with that in the control group *(p* < 0.001). However, the pretreatment with *C. proximus* oil significantly reduced the elevated CVF levels induced by isoproterenol by 66% (*p* = 0.006) (Figure 4).

### 2.6. Effect of *C. Proximus* Oil and/or Isoproterenol on the Level of Fibrosis Markers

**Figure 5:**
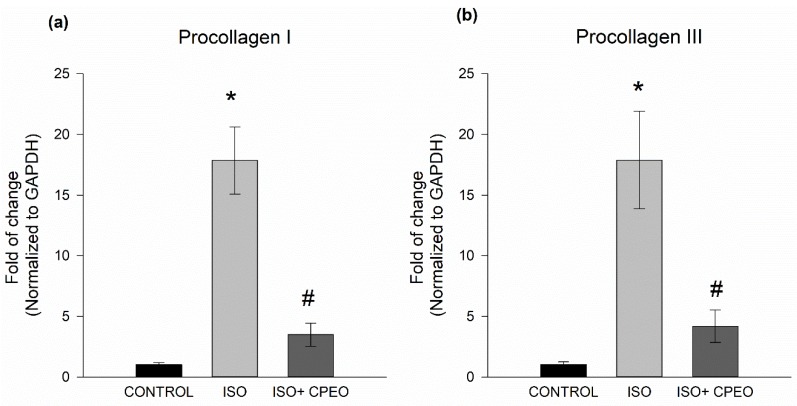
Effect of *Cymbopogon proximus* essential oil (CPEO) and/or isoproterenol (ISO) on levels of fibrosis markers. Male albino rats were injected intraperitoneally daily with vehicle, CPEO (800 µL/kg/d), ISO (5 mg/kg), or CPEO (800 µL/kg/d) plus ISO (5 mg/kg). Oil administration was started four days prior to ISO administration and continued concurrently thereafter for an additional 3 d. Gene expression levels of fibrosis markers Pro I (**a**) and Pro III (**b**) were determined in the heart using quantitative real-time polymerase chain reaction. The results are presented as the means of six independent experiments ± SEM. * *p* < 0.05 compared to control, # *p* < 0.05 compared to ISO-treated rats.

To further assess the extent of changes in myocardial fibrosis mediated by *C. proximus* oil and/or isoproterenol, we measured mRNA levels of fibrotic markers Pro I and Pro III. Isoproterenol treatment resulted in significant induction of Pro I and Pro III expression with 17.8-fold (*p* < 0.001) and 17.9-fold increases (*p* = 0.004), respectively. However, these increases of Pro I and Pro III mRNA levels were significantly reduced by 80% (*p* < 0.001) and 77% (*p* = 0.004), respectively, when the rats were pretreated with *C. proximus* oil (Figure 5).

## 3. Discussion

The results of the present study provide the first evidence that *C. proximus* may confer cardioprotection against cardiac remodeling. Despite advances made in cardiovascular research over the last decades, therapeutic options available for the treatment for HF are limited to agents that either delay disease progression such as β-blockers or only control symptoms such as diuretics [28]. Hence, there is an urgent need to identify new therapeutic agents that either prevent the initiation of HF in high risk patients or regress cardiac hypertrophy during its progression [29]. Over the years, plants have been highly valued around the world as a rich source of therapeutic agents for the treatment and prevention of numerous diseases and illnesses. It is estimated that 80% of cardiovascular drugs are derived from plant origins [30,31]. However, to the best of our knowledge, there has been no research conducted to investigate the cardioprotective effect of *C. proximus* against cardiac remodeling. Therefore, the current study was performed to examine the capacity of *C. proximus* to protect rats from isoproterenol-induced cardiac hypertrophy and fibrosis.

Our study revealed cardioprotective effects of *C. proximus* against isoproterenol-induced cardiac hypertrophy and fibrosis. These findings are evidenced by the prevention of increased HW/BW ratios caused by the administration of isoproterenol to rats pretreated with *C. proximus* oil, which maintained ratios close to those of the control group. In addition, *C. proximus* precluded elevated levels of hypertrophy markers caused by isoproterenol treatment as demonstrated through significant reduction in mRNA levels of ANP, BNP, and β-MHC. Moreover, isoproterenol treatment caused deterioration in cardiomyocyte architecture and increased cell surface area. However, *C. proximus* attenuated these observed effects when administrated prior to the administration of isoproterenol. Histological analysis revealed that isoproterenol treatment induced fibrosis by increasing collagen deposition in the heart. The induction of CVF by isoproterenol was significantly prevented in the group of animals pretreated with *C. proximus* oil, which indicated *C. proximus* had the ability to protect the heart from myocardial fibrosis, a hallmark of cardiac remodeling. Furthermore, isoproterenol-induced elevated mRNA levels of fibrosis markers, including Pro I and Pro III, were significantly reversed by pretreatment with *C. proximus* oil. The dose of *C. proximus* used in our study was chosen based on the study of El Tahir et al. [24]. In their study, *C. proximus* oil causes significant changes in the heart rate only after the administration of the oil at a higher dose (1600 µL/kg). However, 800 µL/kg did not cause significant changes in the heart rate [24]. Therefore, it is unlikely that the effects observed in our study are due to heart rate changes. In addition, *C. proximus* has been shown to exhibit a hypotensive effect in normotensive rats and protect against (L-NAME)-induced hypertension [23,24]. However, it is evident that repeated administration of small doses of isoproterenol to animals causes cardiac hypertrophy and fibrosis without changing the blood pressure [32,33,34]. Thus, it is highly unlikely that *C. proximus* acted as an antihypertensive agent in the absence of hypertensive stimuli in our study. Interestingly, several species of *Cymbopogon* are reported to possess cardiovascular benefiting properties. For instance, extracts of *C. citratus* have been shown to protect against isoproterenol-induced cardiotoxicity [35]. Moreover, extracts from *C. citratus* and *C. winterianus* are shown to reduce blood pressure by modulating the calcium pathway and decreasing heart rate by activating cardiac muscarinic receptors [36,37]. Also, *C. citratus* and *C. jwarancusa* extracts are reported to possess antioxidant, antidiabetic, and hypolipidemic properties and protect against endothelial dysfunction [10,11,12,17,18,21]. Of specific interest, *C. proximus* extracts are reported to possess profound antioxidant effects and are able to decrease blood pressure in both normotensive and hypertensive rats [21,23,24]. Although the chemical compositions of these Cymbopogon species vary, they share some components. For instance, the essential oils of *C. proximus* and *C. jwarancusa* contain a considerable amount of piperitone, carene, β-caryophyllane, and elemol. *Cymbopogon citratus* and *C. winterianus* contain high amounts of geraniol, geranial, and cadinol isomers. Considerable amounts of elemol and limonene have also been reported in both *C. proximus* and *C. winterianus* [11,38,39,40]. Our present findings, along with the results of previous studies, highlight the potential protective effects of Cymbopogon species against CVDs.

Several molecular responses and molecules are well documented to play pivotal roles in the development of cardiac dysfunction and hypertrophy. These include, but are not limited to, inflammatory cytokines, matrix metalloproteinase, oxidative stress, and apoptosis [41,42]. Based on results obtained from GC-MS analysis, the crude *C. proximus* essential oil was comprised of various components that ranged in volume from 0.105% to 23.54%. These findings are consistent with an analysis previously reported [24]. Interestingly, some of the components identified are reported to exhibit various effects on the aforementioned signaling molecules of cardiac hypertrophy. For instance, thymol is reported to protect the heart against isoproterenol-induced myocardial infarction and cardiac hypertrophy via anti-apoptotic effect [43]. In addition, elemol, β-elemene, terpinolene, β- caryophyllene, and thymol, which represented more than 33% of the total essential oil, are known to suppress several pro-inflammatory cytokines, including TNF-α, IL-1β, and IL-6 [44,45,46,47,48]. In addition, production of pro-inflammatory cytokines IL-4, IL-8, and IL-12 was inhibited by elemol, thymol, and β-elemene, respectively [49,50,51]. Moreover, β-caryophyllene decreased the production of matrix metalloproteinases MMP-3 and MMP-9 and the pro-apoptotic markers Bax, p53, and active caspase-3 [45,52,53]. In addition, the major *C. proximus* essential oil extract constituents α-eudesmol and β-eudesmol protect cells from apoptosis by increasing levels of antioxidant enzymes. These pathways counteract the effects of free radicals by decreasing NADPH oxidase and the production of superoxide [54,55]. Modulations of these pathways using genetic approaches and/or pharmacological interventions are shown to be protective against cardiac dysfunction [41,42]. These findings suggest a possible mechanism by which *C. proximus* and its constituents may have produced the protective effects reported in our current study. Identifying the major active constituents of *C. proximus* essential oil, along with the potential mechanisms responsible for the protective effect, requires additional investigation.

## 4. Materials and Methods

### 4.1. Chemicals and Reagents

Isoproterenol was obtained from Sigma-Aldrich (St. Louis, MO, USA) and TRIzol reagent was purchased from Invitrogen Co. (Grand Island, NY, USA). The High-Capacity cDNA Reverse Transcription Kit (Catalog# 4368814) and SYBR^®^ Green PCR Master Mix (Catalog# 4309155) were purchased from Applied Biosystems (Foster City, CA, USA). Hematoxylin and eosin (H&E) and Masson’s trichrome staining kits were purchased from Nanjing SenBeiJia Biological Technology Co., Ltd. (Nanjing, China). Real-time polymerase chain reaction (PCR) primers were designed by members of our laboratory and synthesized by Integrated DNA Technologies Incorporation (San Diego, CA, USA). The primer sequences are shown in Table 2.

### 4.2. Plant Material

*C. Proximus* (Hochst. ex A. Rich.) Stapf, family *Poaceae* was purchased from a local market in Alexandria, Egypt. The identity of the plant material was confirmed by Prof Saniya Kamal at the Department of Botany, College of Science, Alexandria University, Alexandria, Egypt.

### 4.3. Preparation of *C. Proximus* Oil

Essential oil was prepared from dry powdered *C. proximus* plant material (250 gm) using a hydrodistillation method for a period of 5 h [56]. The essential oil was separated and dried over anhydrous sodium sulphate, which yielded a 5.4% *w*/*w* final product.

### 4.4. GC/MS Analysis

GC/MS analysis was carried out using an Agilent 7890 Gas Chromatograph (Agilent, Santa Clara, CA, USA) with an MSD System equipped with a HP-5MS capillary column (30 m × 0.25 mm i.d., 0.25 μm coating). Aliquots (1 mL) of *C. Proximus* oil diluted to a concentration of 5 parts per million (ppm) were then injected into the GC/MS autosampler using the split-less mode. The column temperature was maintained at 70 °C for 5 min and programmed to then increase at a rate of 5 °C/min to 290 °C, which was isothermally held for 5 min. The detector and injector temperatures were 290 °C and 280 °C, respectively. The carrier gas was helium (99.999% purity) at a flow rate of 1.0 mL/min. The significant quadrupole mass analyzer (QMS) operating parameters included electrospray ionization at 70 eV with a scan mass range of 30 to 600 *m*/*z*. The *C. proximus* oil components were identified by comparing their mass spectra with the National Institute of Standards and Technology (NIST 2017) database. The analysis and processing of the results were controlled using MassHunter software (Agilent Technologies Inc., Santa Clara, CA, USA). The identity of peaks was verified by comparing their mass spectra against commercially available libraries (Wiley GC/MS Library, MassFinder 3 Library) as previously described [57,58].

### 4.5. Gas Chromatography (GC) Analysis

GC spectra obtained under the conditions described above were used to identify each peak by comparing their respective relative retention index (RRI) to a series of n-alkanes. The quantity of each compound was estimated based on computerized peak area measurements.

### 4.6. Animals

The study complied with the Law of Ethics of Research on Living Creatures published by the National Committee of BioEthics (NCBE), Saudi Arabia and the National Institutes of Health Guide for the Care and Use of Laboratory Animals (NIH Publications No. 8023, revised 1978). All experimental procedures involving animals were approved by the Bioethics Committee, Prince Sattam Bin Abdulaziz University (No. 201902003). Male albino rats weighing 200–250 g were obtained from the Lab Animal Care Unit, Pharmacy College, Prince Sattam Bin Abdulaziz University (Al-Kharj, KSA). All animals were housed on a 12-h light/dark cycle with food and water available ad libitum.

### 4.7. Experimental Design and Treatment Protocol

Male albino rats were randomly divided into four groups (6 rats/group). The first group received a daily intraperitoneal (IP) injection of vehicle (saline + olive oil). The second group received a daily IP injection of *C. proximus* oil (800 µL/kg/d) with the dose being based on a previous report [24]. The third group received a daily IP injection of isoproterenol (5 mg/kg/d). The fourth group received a daily IP injection of both isoproterenol (5 mg/kg/d) and *C. proximus* oil (800 µL/kg/d). The administration of oil was started four days prior to the isoproterenol administration and continued concurrently thereafter for an additional 3 d. The dose and period of isoproterenol administration were selected based on our previous study [8]. All animal groups were euthanized 24 h after the last dose of treatment. Hearts were quickly excised, washed with saline, blotted with filter paper, and measured, followed by immediately being frozen in liquid nitrogen. The hearts were stored at −80°C until further analysis.

### 4.8. Histological Examination

For histological examinations, heart cross-sections were immediately collected after sacrificing the animals and fixed in 4% formalin at room temperature. The tissues were embedded with paraffin and cut into 3-μm thick sections. The tissue sections were then deparaffinized with xylene and rehydrated with graded ethanol prior to histological staining. For structural analysis, hear tissue sections were stained with H&E using a standard protocol. Images were obtained using a Leica SCN400 Slide Scanner (Leica Biosystems, Wetzlar, Germany) at 200 × magnification. The images were then analyzed using Leica SCN400 Image Viewer software. Random microscopic fields of sections from each animal were selected for analysis. Cell surface area (CSA) of randomly selected cardiomyocytes (10–15 per section) was measured using ImageJ software (National Institute of Health, Bethesda, MD, USA). To visualize and measure collagen deposits, heart tissue sections were stained with Masson’s trichrome according to standard methods. Fibrous tissue stained blue, cytoplasm red, and the cell nuclei black. Cardiac fibrosis was visualized at 200 × magnification using the Leica SCN400 Slide Scanner and the images analyzed using the Leica SCN400 Image Viewer software. CVF was quantified by calculating the area percentage of collagen staining using ImageJ software.

### 4.9. RNA Extraction and Complementary DNA (cDNA) Synthesis

Total RNA was isolated from the frozen tissues using TRIzol reagent according to the manufacturer’s instructions and quantified by measuring absorbance at 260 nm using a Genova Nano micro-volume spectrophotometer (Jenway^®^, Staffordshire, UK). Purity of the RNA was determined according to 260/280 absorbance ratios (>1.8). First strand cDNA was synthesized using a High- Capacity cDNA Reverse Transcription Kit, according to the manufacturer provided instructions. Briefly, 1.5 µg of total RNA from each sample was added to a mixture of 2.0 µL 10× reverse transcriptase buffer, 0.8 µL 25× dNTP mix (100 mM each), 2.0 µL 10× reverse transcriptase random primers, 1.0 µL MultiScribe reverse transcriptase, and 4.2 µL nuclease-free water. The final reaction mixture was maintained at 25 °C for 10 min, heated to 37 °C for 120 min, heated to 85 °C for 5 min, and finally cooled to 4 °C.

### 4.10. Quantification of mRNA Expression by Quantitative Real-Time PCR

Quantitative analysis of specific mRNA expression was performed using real time-PCR. Briefly, 1.5 µg cDNA was subjected to PCR amplification using 96-well optical reaction plates in an ABI Prism 7500 System (Applied Biosystems, Foster City, CA, USA) according to the manufacturer’s protocol. The 25-µL PCR reaction mixture contained 0.25 µL 10-µM forward primer and 0.25 µL 10-µM reverse primer (100 nM final concentration of each primer), 12.5 µL SYBR Green Universal Master Mix, 10.6 µL nuclease-free water, and 1.4 µL cDNA as template. Rat primer sequences for atrial natriuretic peptide (ANP), brain natriuretic peptide (BNP), β-myosin heavy chain (β-MHC), procollagen I (Pro I), procollagen III (Pro III), and glyceraldehyde 3-phosphate dehydrogenase (GAPDH) are listed in Table 2. The real- time PCR data was analyzed as relative gene expression using the 2–∆∆Ct method as previously described [59]. Briefly, the fold change in levels of target genes between the treated and untreated groups were normalized to the level of GAPDH and compared according to the following equation: fold change = 2−∆ (∆Ct), where ∆Ct = Ct(target) − Ct(GAPDH) and ∆ (∆Ct) = ∆Ct(treated) − ∆Ct(untreated).

### 4.11. Statistical Analysis

Statistical analysis of the results from the different experimental groups was performed using SigmaPlot^®^ for Windows (Systat Software, Inc, CA, USA). All data are expressed as means ± SEM. One- way analysis of variance (ANOVA) followed by the Tukey–Kramer multiple comparison test was conducted to assess significant differences between treatment groups. Duplicate reactions were performed for each experiment and the results are presented as the means of six independent experiments ± S.E.M. The differences were considered statistically significant when *p* < 0.05.

## 5. Conclusions

Our study revealed the cardioprotective effects of *C. proximus* essential oil against isoproterenol- induced cardiac hypertrophy and fibrosis. These findings were evidenced by first, significant decreases in HW/BW ratios; second, significant decreases of hypertrophy markers ANP, BNP, and β-MHC mRNA levels; third, significant decreases of fibrosis markers Pro I and Pro III mRNA levels; and fourth, significant decreases in CVF and the inhibition of cardiomyocyte architecture deterioration caused by isoproterenol. Together, these findings pinpoint the importance of *C. proximus* as a potential treatment for cardiac diseases. While the cardioprotective effects of *C. proximus* essential oil were clear, the current findings lack details regarding the correlation between pure components of the essential oil extract and the observed effects. This limitation may be addressed in a future study.

## Figures and Tables

**Table 1:** Components of *Cymbopogon proximus* essential oil.

No.	Component Name	Yield % ^1^
1	Elemol	23.54
2	Piperitone	19.86
3	β-Eudesmol	11.35
4	α-Eudesmol	7.63
5	β-Elemene	4.61
6	τ-Cadinol	3.87
7	Terpinolene	3.48
8	β-Selinenol	2.55
9	3-Cyclohexen-1-one, 2-isopropyl-5-methyl	2.44
10	4-Carene	1.66
11	Shyobunol	1.46
12	α-Terpineol	1.21
13	Cadina-1(10),4-diene	1.13
14	(−)-Guaia-6,9-diene	0.75
15	Limonene	0.66
16	Terpinolene	0.56
17	β-Caryophyllane, 4,8-epoxy	0.52
18	cis-Calamenene	0.51
19	trans-Geranylgeraniol	0.49
20	Epi-Cubenol	0.49
21	Espatulenol	0.49
22	2-Carene	0.44
23	Cuparene	0.38
24	Thymol	0.30
25	(Z)-β-ocimene	0.29
26	Ermacrene B	0.26
27	α-Dihydroagarofuran	0.26
28	γ-Muurolene	0.26
29	Caryophyllene oxide	0.24
30	Shyobunol	0.23
31	α-Selinene	0.20
32	Espatulenol	0.19
33	*p*-Mentha-1,5-dien-8-ol	0.15
34	Anethole	0.14
35	Cadinene	0.13
36	Aromandendrene	0.12
37	δ-Elemene	0.12
38	Isocaryophyllene	0.12
39	Allo-Ocimene	0.11
40	α-Amorphene	0.11
	Total	93.27

^1^ Percentages of yield were calculated based on concentrations obtained according to gas chromatography using an HP-5MS capillary column. The quantitative estimation of each compound was determined based on computerized peak area measurements.

**Table 2:** Sequences of primers used for real-time polymerase chain reaction.

Gene	Forward Primer (5′–3′)	Reverse Primer (5′–3′)
ANP ^a^	GCTTCGGGGGTAGGATTGACA	GGCAATGCGACCAAGCTGT
BNP ^b^	TTTCCTTAATCTGTCGCCGCT	CTAAAACAACCTCAGCCCGTCA
β-MHC ^c^	GCCGAGTCCCAGGTCAACAA	GTAATTCGAGGGCAGGAACCC
Pro I ^d^	CGGCTCCTGCTCCTCTTAGG	CACTCGCCCTCCCGTTTTTG
Pro III ^e^	TGGGATGCAACTACCTTGGT	AGGTGTAGAAGGCTGTGGAC
GAPDH ^f^	CAGTGCCAGCCTCGTCTCAT	CAAGAGAAGGCAGCCCTGGT

^a^ atrial natriuretic peptide; ^b^ brain natriuretic peptide; ^c^ β-myosin heavy chain; ^d^ procollagen I; ^e^ procollagen III; ^f^ glyceraldehyde 3-phosphate dehydrogenase.
